# A Sequential Design for Extreme Quantile Estimation Under Binary Sampling

**DOI:** 10.3390/e28040479

**Published:** 2026-04-21

**Authors:** Michel Broniatowski, Emilie Miranda

**Affiliations:** Laboratoire de Probabilités, Statistique et Modélisation (LPSM), CNRS UMR 8001, Sorbonne Universite, 75005 Paris, France; emilie-miranda@hotmail.fr

**Keywords:** extreme quantile estimation, sequential design, binary information, splitting, extreme value theory

## Abstract

We propose a sequential design method aiming at the estimation of an extreme quantile based on a sample of binary data corresponding to peaks over a given threshold. This study is motivated by an industrial challenge in material reliability and consists of estimating a failure quantile from trials whose outcomes are reduced to indicators of whether the specimen has failed at the tested stress levels. The proposed approach relies on a splitting strategy that decomposes the target extreme probability into a product of higher-order conditional probabilities, enabling a progressive exploration of the tail of the distribution through sampling under truncated laws. We consider GEV and Weibull models for the underlying distribution, and the sequential estimation of their parameters is carried out using an enhanced maximum likelihood procedure specifically adapted to binary data, addressing the substantial uncertainty inherent to such limited information.

## 1. Introduction

Consider a non-negative random variable *X* with distribution function *G*. Let $X_{1},\ldots ,X_{n}$ be *n* independent copies of X. The aim of this paper is to estimate $q_{1-\alpha }$, the $\left(1-\alpha \right)$-quantile of *G* when α is much smaller than 1/n. We therefore aim at the estimation of so-called extreme quantiles. This question has been handled by various authors, and these results are reviewed in Section 3. The approach that we develop is quite different since we do not assume that the $X_{i}$’s can be observed. For any threshold *x*, we define the following random variable:

$$Y=\left\{\begin{array}{c} 1\;\mathrm{if}\;X\leq x\\ 0\;\mathrm{if}\;X>x \end{array}\right.$$

which therefore has a Bernoulli distribution with parameter G(x). The threshold *x* is chosen by the experimenter, but the underlying value of *X* remains unobserved, leading to a substantial loss of information. Such settings are common in industrial statistics. For instance, when assessing the strength of a material or a component, one applies a load *x* and records only whether failure occurs.

In the following, we denote by *R* the resistance of this material and observe the corresponding indicator *Y*. Inference on *G* can be performed for large *n*, making use of many thresholds x. Unfortunately, such a procedure will not be of any help for extreme quantiles.

Indeed, this setting raises significant methodological challenges. First, the observation of binary responses only leads to a substantial loss of information compared to standard settings where the variable of interest is fully observed. Second, the estimation of extreme quantiles, associated with very small probability levels, further exacerbates this difficulty.

Existing approaches for quantile estimation from binary data, in particular those based on adaptations of the Robbins–Monro procedure, typically rely on a precise prior specification of the distribution of the latent variable, including in the vicinity of the target quantile. Such assumptions may be difficult to justify in practice, especially in industrial contexts where prior information is limited. Moreover, these methods are generally designed for moderate probability levels and do not naturally extend to the estimation of extreme quantiles under severe information constraints.

This motivates the development of alternative approaches that are able to explore the tail of the distribution in a progressive manner while reducing the reliance on strong prior assumptions.

To address these limitations, we will consider a design of experiments that progressively explores increasingly extreme regions of the distribution. More precisely, we assume that observations may be collected not only when *R* follows *G*, but also when *R* follows the conditional law of *R* given {R>x}. Under such an assumption, it becomes possible to estimate $q_{1-\alpha }$ even when α<1/n, where *n* denotes the total number of trials.

In materials science, this amounts to considering trials based on artificially modified materials; when estimating extreme upper quantiles, this corresponds to strengthening the material. We would consider a family of increasing thresholds $x_{1},\ldots ,x_{m}$ and for each of them realize $K_{1},\ldots ,K_{m}$ trials, each block of i.i.d. realizations *Y*’s, therefore, corresponds to functions of unobserved *R*’s with distribution *G* conditioned upon $\left\{R>x_{l}\right\}$, 1≤l≤m.

This setting departs significantly from classical approaches based on full data, and is particularly suited for industrial statistics and reliability studies in materials science. From a statistical viewpoint, the situation is tractable when *G* belongs to a parametric family for which the conditional law of *R* given {R>x} preserves the functional form of *G*, up to changes in the parameters. In this case, sampling under conditional laws may be carried out adaptively through a sequential choice of the thresholds $x_{l}$’s. This leads to a recursive estimation procedure in which the parameters of *G* are updated iteratively, and the target quantile $q_{1-\alpha }$ is obtained by combining quantiles of conditional distributions. This splitting-type approach guides the selection of the $x_{l}$’s so that $q_{1-\alpha }$ can ultimately be determined from the final conditional distribution associated with threshold $x_{m}$.

Such techniques are closely connected to safety analysis and pharmaceutical dose-finding, where interest naturally focuses on the behavior of the system under very small probability levels. Through a simple change of variable, these situations can be reframed in terms of upper-tail events. In particular, if $\tilde{R}=1/R$, then for x>0, the event {R<x} is equivalent to $\left\{\tilde{R}>1/x\right\}$. Accordingly, we make use of this duality throughout the paper, exploiting standard results for $\tilde{R}$ when necessary. In particular, if $q_{\alpha }$ denotes the α-quantile of *R* and ${\tilde{q}}_{1-\alpha }$ the (1−α)-quantile of $\tilde{R}$, then $q_{\alpha }=1/{\tilde{q}}_{1-\alpha }$. While this notation may appear slightly cumbersome, it is natural in industrial statistics.

This article is organized as follows, and its main contributions are introduced along the way. Section 2 formalizes the problem in the framework of an industrial application and highlights the specific challenges arising from binary observations and extreme quantile estimation. Section 3 reviews the existing literature on extreme quantile estimation and experimental design under binary data. This discussion emphasizes the limitations of current approaches in the regime of very small probabilities and motivates the need for alternative strategies. In Section 4, we introduce a novel splitting-based framework for extreme quantile estimation under partial observability. This constitutes a central contribution of the paper, as it enables the progressive exploration of the tail of the distribution through a sequential experimental design. The proposed procedure is elaborated for a Generalized Pareto model. Section 5 develops the associated estimation procedure. In particular, we propose a dual-criterion approach combining a likelihood-based component with a stability condition on sequentially estimated quantiles. Section 6 extends the methodology to an alternative parametric model, illustrating the flexibility of the proposed framework beyond the Generalized Pareto setting. Finally, Section 7 and Section 8 provide a brief discussion of model selection and behavior under misspecification, as well as hints about extensions of the models studied beforehand.

## 2. Problem Formulation in an Industrial Reliability Framework

This study focuses on the estimation of extreme failure quantiles, a critical issue in industrial risk assessment. Such quantiles play a major role in engineering applications, particularly in the aeronautics industry, where they intervene directly in decisions pertaining to engine component dimensioning and the management of fatigue-related risks. For a detailed presentation of the industrial context related to material fatigue that motivated this study, see Broniatowski and Miranda (2019) [1].

In this context, the usual estimation procedures rely on data obtained from experimental trial campaigns, in which specimens are subjected to various stress levels and tested until failure or until the end of the trial. Such experimental campaigns can be extremely costly in certain industrial contexts, such as aeronautics, which severely limit both the total number of trials and the diversity of experimental conditions that can be explored.

The aim of this study is to introduce a new experimental design methodology for the estimation of extreme failure quantiles at a fixed target lifetime, under a very low risk level α of the order of $10^{-3}$, while relying on a minimal number of trials.

Let *S* denote the stress level applied (in MPa).

Throughout the paper, we will denote *R*, a positive r.v. modeling the resistance of the material at the target lifetime and homogeneous to the stress.

Define $s_{\alpha }$ as the failure quantile of probability level α$=10^{-3}$, the level of stress that guarantees that the risk of failure before the fixed lifetime does not exceed α. Thus, $s_{\alpha }$ is the α-quantile of the distribution of *R*:

(1) $$s_{\alpha }=q_{\alpha }=\sup\left\{s:P(R\leq s)\leq \alpha \right\}.$$

However, *R* is not directly observed during the experiments. The relevant information available to characterize *R* is limited to indicators of whether or not the specimen tested has failed at *s* before the end of the trial. Therefore, the relevant observations corresponding to a campaign of *n* trials are formed by a sample of variables $Y_{1},\ldots ,Y_{n}$ with for 1≤i≤n,

$$Y_{i}=\left\{\begin{array}{c} 1\;\mathrm{if}\;R_{i}\leq s_{i}\\ 0\;\mathrm{if}\;R_{i}>s_{i} \end{array}\right.$$

where $s_{i}$ is the stress applied on specimen i.

Note that the number of observations is constrained by industrial and financial considerations. Thus, α is way lower than 1/n, and we are considering a quantile lying outside the sample range.

Although this work is motivated by an industrial application in materials science, similar statistical settings arise in other fields, such as broader reliability analysis or dose-finding studies in medical trials, where the goal is to estimate a maximum tolerated dose corresponding to a very small failure probability.

## 3. Extreme Quantile Estimation—A Short Survey

As seen above, estimating extreme failure quantiles raises two main issues: on the one hand, the estimation of an extreme quantile, and on the other hand, the need to conduct inference based on exceedances under thresholds. We provide here a brief overview of these two areas, bearing in mind that the literature on extreme quantile estimation typically assumes complete data or, at best, right-censored observations.

### 3.1. Extreme Quantiles Estimation Methods

Extreme quantile estimation in the univariate setting is widely covered in the literature when the variable of interest *X* is either completely or partially observed. The usual framework is the study of the (1−α)-quantile of a r.v *X*, with very small alpha.

The most classical case corresponds to the setting where $x_{1-\alpha }$ is drawn from an *n* sample of observations $X_{1},\ldots ,X_{n}$. A distinction is usually made between high quantiles that lie within the sample range (see Weissman 1978 [2] and Dekkers et al., 1989 [3]) and extreme quantiles that fall outside the range of the observations (e.g., De Haan and Rootzén 1993 [4]). It is assumed that *X* belongs to the domain of attraction of an extreme value distribution. The tail index of the latter is then estimated through maximum likelihood (Weissman 1978 [2]) or through an extension of Hill’s estimator, such as the moment estimator by Dekkers et al. (1989) [3]. The estimator of the quantile is then deduced from the inverse function of the distribution of the *k* largest observations. Note that all the above references assume that the distribution has a Pareto tail.

Alternative modeling strategies have been proposed by De Valk (2016) [5] and De Valk and Cai (2018) [6], who assume a Weibull-type tail. This relaxes certain second-order hypotheses on the tail and allows one to target quantiles far outside the sample range. We will use these methods as benchmarks in our empirical comparisons.

Recent studies have also tackled the issue of censoring. For instance, Beirlant et al. (2007) [7] and Einmahl et al. (2008) [8] proposed a generalization of the peak-over-threshold method when the data are subjected to random right censoring and an estimator for extreme quantiles. The idea is to consider a consistent estimator of the tail index on the censored data and divide it by the proportion of censored observations in the tail. Worms and Worms (2014) [9] studied estimators of the extremal index based on Kaplan–Meier integration and censored regression.

By contrast, the literature is much sparser in the case of complete truncation, i.e., when only exceedances over predetermined thresholds are observed. All of the methods mentioned above rely on the higher-order statistics of the original sample, which are not available in the present setting. Consequently, classical extreme quantile estimators are not directly applicable to our problem.

In the next subsection, we therefore turn to sequential experimental designs used in industrial and biostatistical contexts, and assess their relevance for estimating extreme quantiles from dichotomous data.

### 3.2. Sequential Design Based on Dichotomous Data

A few studies have addressed the estimation of quantiles from binary data. Wu (1985) [10] and Joseph (2004) [11] propose adaptations of the Robbins and Monro procedure for binary data. Yet both focus on quantiles of moderate order (typically between 0.1 and 0.9) and require a fairly accurate prior knowledge of the latent distribution of the variable of interest around the targeted quantile. Such an assumption may be unrealistic in many applications, especially when targeting the tail of the distribution.

Wu and Tian (2014) [12] introduced what appears to be the most advanced procedure for quantile estimation based on binary data. Their three-step sequential approach (search, estimate, approximate) shows promising performance even for extreme quantiles, but it relies heavily on the specifics of the application context and must be tailored on a case-by-case basis.

In contrast, the approach we propose in this study aims to provide a generic framework specifically designed for the estimation of extreme failure quantiles. Our methodology is grounded in parametric assumptions justified by extreme value theory results, thus reducing the need for strong prior knowledge on the latent distribution and reducing sensitivity to model specification.

The remainder of this section reviews two classical designs frequently used in industry and biostatistics and conceptually closest to our objective: the *Staircase* and the *Continual Reassessment Method (CRM)*. In what follows, we consider the estimation of a small quantile $q_{\alpha }$ for events of the form {R<s} with α small. Both procedures rely on a parametric model for the strength variable *R*. We retain two specifications to facilitate performance comparisons, rather than to provide fully realistic safety assessments.

#### 3.2.1. The Staircase Method

Denote $P\left(R\leq s\right)=\phi \left(s,\theta_{0}\right)$. Invented by Dixon and Mood (1948) [13] and refined in Dixon (1965) [14], this technique aims at the estimation of the parameter $\theta_{0}$ through sequential search based on data of exceedances under thresholds. Starting from an initial stress level $S_{ini}$, each item is tested and the next test level is adjusted by a fixed increment δ>0: it is increased after a survival and decreased after a failure. This process is repeated for the *K* specimens. After the *K* trials, the parameter $\theta_{0}$ is estimated through maximization of the likelihood.

The proper conduct of the Staircase method relies on strong assumptions about the choice of the design parameters. Firstly, $S_{ini}$ has to be sufficiently close to the expectation of *R* and secondly, δ has to lie between 0.5σ and 2σ, where σ designates the standard deviation of the distribution of *R*.

##### Numerical Results

The accuracy of the procedure has been evaluated on the two models presented below on a batch of 1000 replications, each with K=100.

*Exponential case*

Let R∼E(λ) with λ=0.2. The input parameters are $S_{\mathrm{ini}}=5$ and $\delta =15\in \left[0.5\times \frac{1}{\lambda^{2}},2\times \frac{1}{\lambda^{2}}\right]$.

As shown in Table 1, the relative error pertaining to the parameter λ is roughly 25%, although the input parameters are reasonably well chosen for the method. The resulting relative error on the $10^{-3}$ quantile is 40%. Indeed, the parameter λ is underestimated, which results in an overestimation of the variance $1/\lambda^{2}$, which induces an overestimation of the $10^{-3}$ quantile.

*Gaussian case*

We now choose R∼N(μ,σ) with μ=60 and σ=10. The value of $S_{\mathrm{ini}}$ is set to the expectation and δ=7 belongs to the interval $\left[\frac{\sigma }{2},2\sigma \right].$ The same procedure as above is performed and yields the results in Table 2.

The expectation of *R* is recovered rather accurately, whereas the estimation of the standard deviation suffers a loss in accuracy, which, in turn, yields a relative error of 180 % on the $10^{-3}$ quantile.

##### Limitations of the Staircase Method

While the Staircase method can recover the central tendency with a limited number of trials, it is not suitable for extreme quantile estimation. The latter relies on extrapolation from potentially biased parameter estimates, and simple reparametrizations (e.g., in terms of the extreme quantile) do not remedy the inherent loss of accuracy.

#### 3.2.2. The Continuous Reassessment Method (CRM)

##### General Principle

The CRM (O’Quigley, Pepe and Fisher, 1990 [15]) has been designed for clinical trials to estimate $q_{\alpha }$ among *J* stress levels $s_{1},\ldots ,s_{J}$, when α is of order 20%.

Denote $P\left(R\leq s\right)=\psi \left(s,\beta_{0}\right)$. The estimator of $q_{\alpha }$ is

$$s^{*}:=\underset{s_{j}\in \left\{s_{1},\ldots ,s_{J}\right\}}{\mathrm{arginf}}\left|\psi (s_{j},\beta_{0})-\alpha \right|.$$

This optimization is performed iteratively with *K* trials per iteration. Starting with an initial estimator $\hat{\beta_{1}}$ of $\beta_{0}$, for example, through a Bayesian choice as proposed in [15], define

$$s_{1}^{*}:=\underset{s_{j}\in \left\{s_{1},\ldots ,s_{J}\right\}}{\mathrm{arginf}}\left|\psi (s_{j},\hat{\beta_{1}})-\alpha \right|.$$

Each iteration i≥1 follows a two-step procedure:

- Update ${\hat{\beta }}_{i}$ using all past data (maximum likelihood or Bayesian posterior under $\psi (\cdot ,\beta_{0})$).
- Set
  $$s_{i}^{*}:=\underset{s_{j}\in \left\{s_{1},\ldots ,s_{J}\right\}}{\mathrm{arginf}}\;\left|\psi \left(s_{j},{\hat{\beta }}_{i}\right)-\alpha \right|,$$
  and perform the next *K* Bernoulli trials at level $s_{i}^{*}$.

The stopping rule depends on the context (maximum number of trials or stabilization of the results).

Note that the Bayesian inference is useful in cases where there is no diversity in the observations at some iterations of the procedure, i.e., when, at a given level of test $s_{i}^{*}$, only failures or survivals are observed.

##### Application to Failure Quantiles

Denote by $\pi_{s}$ the prior indexed by the stress level *s*. $\pi_{s}$ models the failure probability at level *s* and has a Beta distribution given by

(2) $$\pi_{s}∼\beta \left(k,n-k+1\right).$$

It amounts to stating that *at a given stress level s, we expect k failures out of n trials*.

Let *R* follow an exponential distribution: $\forall s\geq 0,\psi \left(s,\beta_{0}\right)=p_{s}=1-\exp\left(-\beta_{0}s\right)$.

It follows $\forall s,\;\beta_{0}=-\frac{1}{s}\log\left(1-p_{s}\right)$.

Define the random variable $\Lambda_{s}=-\frac{1}{s}\log\left(1-\pi_{s}\right)$ which, by definition of $\pi_{s}$, is distributed as a k-order statistic of a uniform distribution $U_{k,n}$.

The estimation procedure of the CRM is obtained as follows:

*Step 1.* Compute an initial estimator of the parameter

$$\Lambda_{s}=\frac{1}{L}\sum_{l=1}^{L}-\frac{1}{s}\log\left(1-\pi_{s}^{l}\right)$$

with $\pi_{s}^{l}∼\beta \left(k,n-k+1\right),1\leq l\leq L$. Define

$$s_{1}^{*}:=\underset{s_{j}\in \left\{s_{1},\ldots ,s_{J}\right\}}{\mathrm{arginf}}\left|(1-\exp\left(-\Lambda_{s}s_{j}\right))-\alpha \right|.$$

and perform *J* trials at level $s_{1}^{*}$. Denote the observations $Y_{1,j}:=1_{R_{1,j}<s_{1}^{*}},\;1\leq j\leq J.$

*Step i.* At iteration *i*, compute the posterior distribution of the parameter:

(3) $$\pi_{s_{i}}^{*}∼\beta \left(k+\sum_{l=1}^{i}\sum_{j=1}^{J}Y_{l,j}\;,\;n+\left(J\times i\right)-\left(k+\sum_{l=1}^{i}\sum_{j=1}^{J}Y_{l,j}\right)+1\right)$$

The above distribution also corresponds to an order statistic of the uniform distribution $U_{k+\sum_{l=1}^{i}\sum_{j=1}^{J}Y_{l,j}\;,\;n+\left(J\times i\right)}$. We then obtain an estimate $\Lambda_{s_{1}^{*}}$.

The next stress level $s_{i+1}^{*}$ to be tested in the procedure is then given by

$$s_{i+1}^{*}:=\underset{s_{j}\in \left\{s_{1},\ldots ,s_{J}\right\}}{\mathrm{arginf}}\left|(1-\exp\left(-\Lambda_{s_{1}^{*}}s_{j}\right))-\alpha \right|.$$

##### Numerical Simulation for the CRM

Under the exponential model with parameter λ=0.2, using N=10 iterations, J=10 equally spaced thresholds $s_{1},\ldots ,s_{J}$, and K=50 trials per iteration, we obtain the results reported in Table 3.

**Figure 1:**
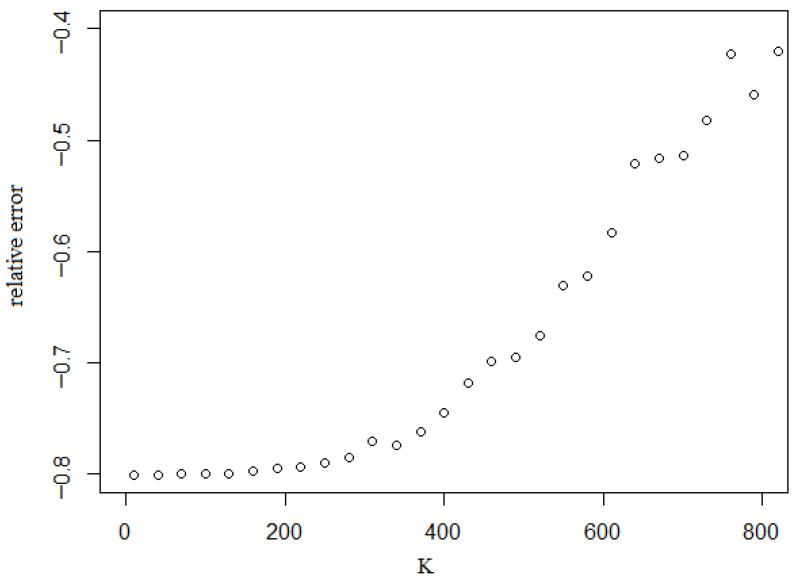
Relative error on the $10^{-3}$-quantile with respect to the number of trials for each stress level.

The $10^{-3}$-quantile is poorly estimated even in this simple setting. Near the target threshold, almost no failures are observed; for acceptable values of *K*, the method becomes ineffective. Figure 1 illustrates the improvement in accuracy as *K* increases.

In summary, both the Staircase and the CRM face the same limitation for extreme quantile estimation. The staircase targets the central tendency, whereas the CRM is calibrated for moderate quantile levels (around 0.2), far from the target $\alpha =10^{-3}$. This motivates the original procedure proposed in the next sections, specifically designed for extreme quantiles under binary information.

## 4. A New Design for the Estimation of Extreme Quantiles

### 4.1. Splitting

The design we propose is directly inspired by the general principle of splitting methods used in the domain of rare events simulation and introduced by Kahn and Harris (1951) [16].

The central idea is to overcome the difficulty of estimating an extremely small probability by decomposing the target event into a sequence of events of higher probability. Splitting achieves this by expressing a rare-event probability as a product of conditional probabilities that are easier to estimate.

Let P denote the distribution of the random variable *R*. The event $\left\{R\leq s_{\alpha }\right\}$ can be expressed as the intersection of inclusive events for $s_{\alpha }=s_{m}<s_{m-1}<\ldots <s_{1}$ it holds:

$$\left\{R\leq s_{\alpha }\right\}=\left\{R\leq s_{m}\right\}\subset \cdots \subset \left\{R\leq s_{1}\right\}.$$

It follows that

(4) $$\alpha =P\left(R\leq s_{\alpha }\right)=P\left(R\leq s_{1}\right)\prod_{j=1}^{m-1}P\left(R\leq s_{j+1}|R\leq s_{j}\right)$$

The thresholds ${\left(s_{j}\right)}_{j=1,\ldots ,m}$ should be chosen so that each conditional probability $P(R\leq s_{j+1}|R\leq s_{j})$ is of moderate level (typically p=0.2 or 0.3). This ensures that the event $\left\{R\leq s_{j+1}\right\}$ is observable under the conditional distribution of $R|R\leq s_{j}$, while the product of these probabilities still reconstructs the target rare-event probability α via (4), with a small number of stages *m*.

From the formal decomposition in (4), a practical experimental scheme can be deduced. Its structure is given in Procedure 1.

**Remark** **1.**  *Our approach can be seen as an alternative to stochastic approximation methods for quantile estimation under binary responses. While these methods rely on recursive updates driven by observed responses, they typically require either moderate quantile levels or strong prior assumptions on the underlying distribution.*

*In contrast, the procedure proposed here combines a sequential design with structural modeling inspired by extreme value theory, allowing the exploration of much more extreme regions of the distribution under weaker assumptions.*

**Remark** **2.**  ***Practical feasibility:*** *The above procedure relies on the assumption that sampling can be performed from the conditional distribution of the resistance at each step. In practice, this amounts to conducting experiments on specimens with progressively reduced resistance levels. Although this assumption may appear strong, it is, in fact, realistic in some experimental settings, as specimens with controlled weakened resistance can be manufactured through appropriate machining processes. An illustrated example of such an approach in the context of material fatigue is provided in Broniatowski and Miranda (2019) [1].*

**Procedure 1** Splitting procedure

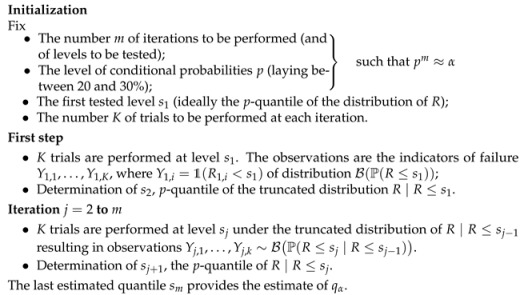



### 4.2. Choice of Sequential Design Parameters

The performance of the proposed sequential procedure depends on several parameters, namely the conditional probability level *p*, the number of stages *m*, and the number of trials per stage *K*. Their selection involves a trade-off between statistical accuracy and experimental cost.

A standard choice for the conditional probability level *p* is to fix it around 20%. This value ensures that the conditional failure probability remains sufficiently large to be reliably estimated from a limited number of binary observations, while still enabling a progressive exploration of the tail of the distribution through the splitting mechanism.

The first threshold $s_{1}$ is selected so as to approximate the target conditional probability level *p*. In practice, however, the resulting probability $p_{1}$ may differ from *p*. In such cases, the model parameters are updated to ensure consistency with the target rare event probability α, typically through the relation

$$p_{1}\cdot p^{m-1}\approx \alpha .$$

The selection of $s_{1}$ may also rely on domain-specific expertise, depending on the application context. In principle, the first iteration could be refined by incorporating additional prior information, for instance, regarding the central tendency of the distribution. However, such extensions are not considered in the present work in order to preserve the generality of the proposed methodology.

More generally, the parameters *p*, *m*, and *K* must be chosen jointly. Increasing the number of stages *m* allows for a more gradual progression toward extreme regions, which may improve robustness and stability, but also increases the overall complexity of the procedure. Increasing the number of trials *K* at each stage improves the accuracy of the parameter estimates and of the conditional quantiles, at the cost of a higher experimental burden. Finally, the choice of *p* controls the balance between these two effects: smaller values of *p* lead to a faster progression toward the tail but reduce the amount of information available at each stage, whereas larger values improve estimation reliability but require more stages to reach the target rare event probability.

In practice, this trade-off is largely driven by application-specific constraints. In industrial settings, for instance, the cost of performing experiments may increase significantly as testing conditions become more severe (corresponding to deeper levels in the sequential procedure), while the availability of resources (time, equipment, personnel) may limit the number of feasible trials. As a result, the parameters *p*, *m*, and *K* are often not freely chosen by the statistician, but rather dictated by these operational constraints. The guidelines provided here should therefore be understood as indicative rather than prescriptive. A detailed illustration of these trade-offs in an industrial context is provided in Broniatowski and Miranda (2019) [1].

### 4.3. Modeling the Distribution of the Strength, Pareto Model

The events under consideration have a small probability under P. By (4), we are led to consider the limit behavior of conditional distributions under smaller and smaller thresholds. To this end, we rely on the classical approximations due to Balkema and de Haan (1974) [17] and Pickands (1975) [18] stated below in the familiar setting of exceedances above high thresholds. Let $\tilde{R}:=1/R$.

**Theorem** **1.**  *For $\tilde{R}$ of distribution F belonging to the maximum domain of attraction of an extreme value distribution with tail index c, i.e., F∈MDA(c), it holds that: there exists a=a(s)>0, such that:*

$$\lim_{s\to \infty }\underset{0\leq x<\infty }{\sup}\left|\frac{1-F\left(x+s\right)}{1-F\left(s\right)}-\left(1-G_{\left(c,a\right)}\left(x\right)\right)\right|=0$$

*where $G_{\left(c,a\right)}$ is defined through*

$$G_{\left(c,a\right)}\left(x\right)=1-\exp\left\{-\int_{0}^{\frac{x}{a}}{\left[{\left(1+ct\right)}_{+}\right]}^{-1}dt\right\}$$

*where a>0 and c∈R.*

The distribution *G* is the Generalized Pareto distribution GPD(c,a) given explicitly by

$$1-G\left(x\right)=\left\{\begin{array}{c} {\left(1+\frac{c}{a}x\right)}^{-1/c}\;\mathrm{when}\;c\neq 0\\ \exp(-\frac{x}{a})\;\mathrm{when}\;c=0 \end{array}\right.$$

where x≥0 for c≥0 and $0\leq x\leq -\frac{a}{c}$ if c<0.

A key feature of GPDs is their invariance under threshold conditioning. Indeed it holds, for $\tilde{R}∼GDP\left(c,a\right)$ and x>s,

(5) $$P\left(\tilde{R}>x|\tilde{R}>s\right)={\left(1+\frac{c(x-s)}{a+cs}\right)}^{-1/c}$$

We therefore state:

**Proposition** **1.**  *When $\tilde{R}∼GPD\left(c,a\right)$ then, given $\left(\tilde{R}>s\right)$, the random variable $\tilde{R}-s$ follows a GPD(c,a+cs).*

Thus, GPDs are both stable under thresholding and arise as limiting models for threshold exceedances. This mirrors the classical rationale that motivates the use of normal or stable laws in additive models. These properties make GPDs particularly suitable to model $\tilde{R}$ for excess-probability inference. Due to the lack of memory property, the exponential distribution, which appears as a possible limit distribution for excess probabilities when c=0 in Theorem 1, does not qualify for modeling. Moreover, since *R* may take arbitrarily small values (i.e., $\tilde{R}$ is unbounded), we restrict attention to c>0.

Turning to the context of extreme failure quantiles, we make use of the random variable $R=1/\tilde{R}$ and proceed to the corresponding change of variable.

When c>0, the distribution function of the r.v. *R* writes for nonnegative *x*:

(6) $$F_{c,a}\left(x\right)={\left(1+\frac{c}{ax}\right)}^{-1/c}.$$

For 0<x<u, the conditional distribution of *R* given $\left\{R<u\right\}$ is

$$P\left(R<x|R<u\right)={\left(1-\frac{c(\frac{1}{x}-\frac{1}{u})}{a+\frac{c}{u}}\right)}^{-1/c}$$

showing that the distribution of *R* is stable under threshold conditioning with parameter $\left(a_{u},c\right)$, where

(7) $$a_{u}=a+\frac{c}{u}.$$

In practice, at step *j* of the procedure, the stress level $s_{j}$ is the corresponding threshold $1/{\tilde{s}}_{j}$, where ${\tilde{s}}_{j}$ is a right-quantile of the conditional law of $\tilde{R}$ given $\left\{\tilde{R}>{\tilde{s}}_{j-1}\right\}$. Therefore, the observations take the form $Y_{i}=1_{R<s_{j}|R<s_{j-1}}=1_{{\tilde{R}}_{i}>{\tilde{s}}_{j}|{\tilde{R}}_{i}>{\tilde{s}}_{j-1}},\;\;i=1,\ldots ,K_{j}$.

A convenient feature of model (6) lies in the fact that the conditional distributions are completely determined by the initial distribution of *R*, therefore by a and c. The parameters $a_{j}$ of the conditional distributions are determined from these initial parameters and by the corresponding stress level $s_{j};$ see (7).

### 4.4. Notation and Framework for Sequential GPD Modeling and Parameterization

We assume that the distribution of the random variable $\tilde{R}$ follows a Generalized Pareto Distribution $GPD(c_{T},a_{T})$ with distribution function $G_{\left(c_{T},a_{T}\right)}$, and denote ${\bar{G}}_{\left(c_{T},a_{T}\right)}=1-G_{\left(c_{T},a_{T}\right)}$.

The proposed procedure relies on a sequence of increasing thresholds $\left({\tilde{s}}_{1},\ldots ,{\tilde{s}}_{m}\right)$ and exploits the stability property of the GPD under threshold exceedances. As stated in (5), for each j∈{1,…,m}, the conditional distribution of $\tilde{R}$ given $\left\{\tilde{R}>{\tilde{s}}_{j}\right\}$ remains a GPD and can be written as

$${\bar{G}}_{\left(c_{j},a_{j}\right)}\left(x-{\tilde{s}}_{j}\right)=P\left(\tilde{R}>x|\tilde{R}>{\tilde{s}}_{j}\right),$$

with $c_{j}=c_{T}$ and $a_{j}=a_{T}+c_{T}{\tilde{s}}_{j}$.

At iteration *j*, we denote by ${\left(\hat{c},\hat{a}\right)}_{j}$ the estimators of $\left(c_{j},a_{j}\right)$, so that $1-G_{{\left(\hat{c},\hat{a}\right)}_{j}}\left(x-{\tilde{s}}_{j}\right)$ provides an estimate of $P(\tilde{R}>x|\tilde{R}>{\tilde{s}}_{j})$. The parameters of the initial distribution $\left(c_{T},a_{T}\right)$ can then be recovered from ${\left(\hat{c},\hat{a}\right)}_{j}$ through

$${\hat{c}}_{T}=\hat{c},\;{\hat{a}}_{T}=\hat{a}-\hat{c}\;{\tilde{s}}_{j}.$$

### 4.5. Sequential Design for the Extreme Quantile Estimation

Fix *m* and *p*, where *m* denotes the number of stress levels under which the trials will be performed, and *p* is such that $p^{m}=\alpha .$

Select an initial stress level $s_{1}$ that is sufficiently large (i.e., ${\tilde{s}}_{1}=1/s_{1}$ sufficiently small) so that $p_{1}=P\left(R<s_{1}\right)$ is large enough and perform trials at this level. Ideally, one would choose $s_{1}$ such that $p_{1}=p$, which cannot be secured; in practice, this choice relies on expert judgment.

Switch to the transformed variable $\tilde{R}:=1/R$. Estimate the GPD parameters $\left(c_{T},a_{T}\right)$ of $\tilde{R}$ based on the observations above ${\tilde{s}}_{1}$, producing the initial estimates ${\left(\hat{c},\hat{a}\right)}_{1}$. As stated in Section 4.2, $s_{1}$ is chosen so that it corresponds to medium stress conditions, and failures are therefore easy to observe experimentally.

Define

$${\tilde{s}}_{2}:=\sup\left\{s:{\bar{G}}_{{\left(\hat{c},\hat{a}\right)}_{1}}\left(s-{\tilde{s}}_{1}\right)<p\right\}$$

the (1−p)-quantile of $G_{{\left(\hat{c},\hat{a}\right)}_{1}}.$${\tilde{s}}_{2}$ is the level of stress to be tested at the following iteration.

Iterating from step j=2 to m−1, perform *K* trials under $G_{\left(c_{j},a_{j}\right)}$ say ${\tilde{R}}_{j,1},\ldots ,{\tilde{R}}_{j,K}$ and consider the observable variables $Y_{j,i}:=1_{{\tilde{R}}_{j,i}>{\tilde{s}}_{j}}$. Therefore, the *K* iid replications $Y_{j,1},\ldots ,Y_{j,K}$ follow a Bernoulli $B({\bar{G}}_{\left(c_{j-1},a_{j-1}\right)}\left({\tilde{s}}_{j}-{\tilde{s}}_{j-1}\right))$, where ${\tilde{s}}_{j}$ has been determined at the previous step of the procedure. Estimate $\left(c_{j},a_{j}\right)$ in the resulting Bernoulli scheme, say ${\left(\hat{c},\hat{a}\right)}_{j}$. Then define

$$\begin{array}{cc} {\tilde{s}}_{j+1} & :=\sup\left\{s:{\bar{G}}_{{\left(\hat{c},\hat{a}\right)}_{j}}\left(s-{\tilde{s}}_{j}\right)<p\right\}\\ & =G_{{\left(\hat{c},\hat{a}\right)}_{j}}^{-1}\left(1-p\right)+{\tilde{s}}_{j}, \end{array}$$

which is the (1−p)-quantile of the estimated conditional distribution of $\tilde{R}$ given $\left\{\tilde{R}>\tilde{s_{j}}\right\}$, i.e., $G_{{\left(\hat{c},\hat{a}\right)}_{j}}$, and the next level to be tested.

In practice a conservative choice for *m* is $m=\left\lceil \frac{log\alpha }{logp}\right\rceil$, where ⌈.⌉ denotes the ceiling function. The attained probability $\tilde{\alpha }$ is a proxy of α (see Section 5).

The *m* stress levels ${\tilde{s}}_{1}<{\tilde{s}}_{2}<\ldots <{\tilde{s}}_{m}={\tilde{q}}_{1-\alpha }$ satisfy

$$\begin{array}{cc} \tilde{\alpha } & =\bar{G}\left({\tilde{s}}_{1}\right)\prod_{j=1}^{m-1}{\bar{G}}_{{\left(\hat{c},\hat{a}\right)}_{j}}\left({\tilde{s}}_{j+1}-{\tilde{s}}_{j}\right)\\ & =p_{1}p^{m-1} \end{array}$$

Finally, by construction, ${\tilde{s}}_{m}$ is a proxy of ${\tilde{q}}_{1-\alpha }$.

Although conceptually simple, the method raises several challenges, chiefly regarding the estimation of ${\left(\hat{c},\hat{a}\right)}_{j}$ at each stage. The next section addresses this issue.

## 5. Enhanced Sequential Design in the Pareto Model

In this section, we focus on the estimation of the parameters $\left(c_{T},a_{T}\right)$ in the $GPD(c_{T},a_{T})$ distribution of $\tilde{R}.$ One of the main difficulties lies in the fact that the available information does not consist of replications of the random variable $\tilde{R}$ under the current conditional distribution $G_{\left(c_{j-1},a_{j-1}\right)}$ of $\tilde{R}$ given $\left(\tilde{R}>\tilde{s_{j-1}}\right)$ but merely on the very downgraded functions of those.

At step *j*, we are given $G_{{\left(\hat{c},\hat{a}\right)}_{j-1}}$ and derive ${\tilde{s}}_{j}$ as its (1−p)-quantile. Simulating *K* random variable ${\tilde{R}}_{j,i}$ with distribution $G_{\left(c_{j-1},a_{j-1}\right)}$, the only observable quantities are the Bernoulli(*p*) variables

$$Y_{j,i}:=1_{{\tilde{R}}_{j,i}>{\tilde{s}}_{j}},$$

which represent a substantial loss of information compared with the underlying ${\tilde{R}}_{j,i}$. Estimating ${\left(\hat{c},\hat{a}\right)}_{j}$ from these $Y_{j,i}$’s is therefore intrinsically challenging.

### 5.1. Limitations of Likelihood-Based Estimation

**Figure 2:**
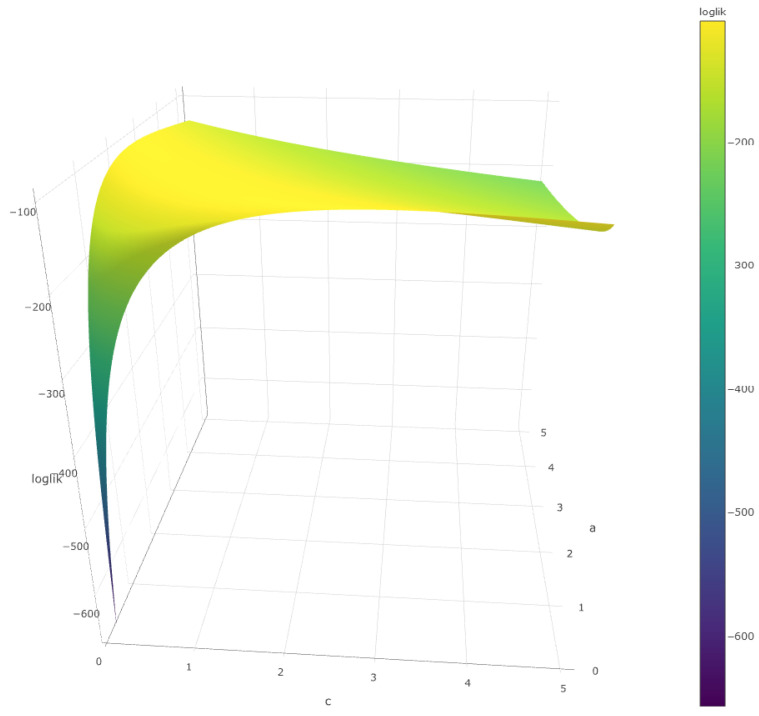
Log-likelihood of the Pareto model with binary data.

A natural approach is to estimate the parameters ${\left(c,a\right)}_{j}$ at each iteration using maximum likelihood. However, this leads to a rapid deterioration of the estimation of the extreme quantile ${\tilde{q}}_{1-\alpha }$ for small α. In simulation experiments, the large standard deviation of ${\hat{\tilde{q}}}_{1-\alpha }$ is directly linked to the poor precision of the iterative estimators ${\left(\hat{c},\hat{a}\right)}_{j}$. To illustrate this, we generated n=200 independent Bernoulli variables $Y_{i}$ with parameter ${\bar{G}}_{\left(c_{T},a_{T}\right)}\left({\tilde{s}}_{1}\right)$. Figure 2 displays the corresponding log-likelihood profile as the Bernoulli parameter ${\bar{G}}_{\left(c^{\prime },a^{\prime }\right)}\left({\tilde{s}}_{0}\right)$ varies with $\left(c^{\prime },a^{\prime }\right)$. As expected, the log-likelihood surface is extremely flat across a wide region of the parameter space.

This explains the poor results in Table 4 obtained through the Splitting procedure when the parameters at each step are estimated by maximum likelihood, particularly the high variability in the estimated quantiles. Moreover, the accuracy of the estimator of ${\tilde{q}}_{1-\alpha }$ quickly decreases with the number *K* of replications $Y_{j,i}$, 1≤i≤K, as illustrated by results in Table 5.

**Figure 3:**
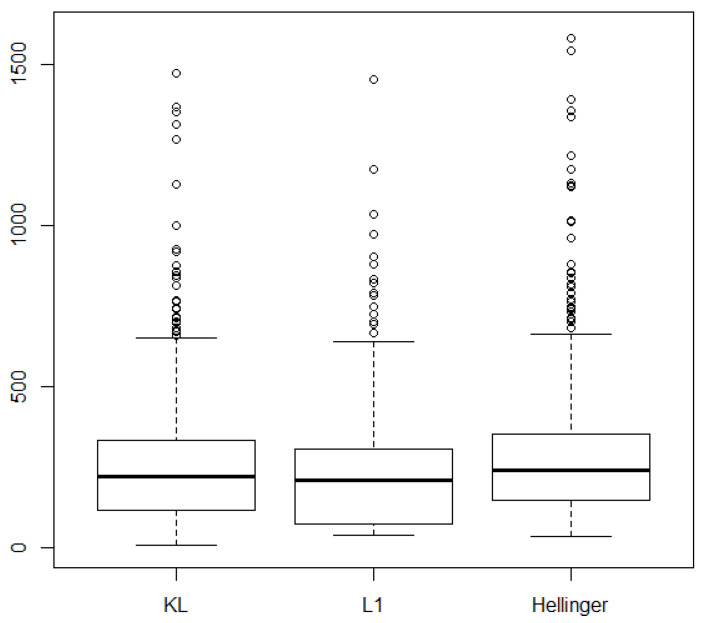
Estimations of the α-quantile based on the Kullback–Leibler, L1 distance, and Hellinger distance criterion.

Changing the estimation criterion by some alternative method does not improve significantly; Figure 3 shows the distribution of the resulting estimators of ${\tilde{q}}_{1-\alpha }$ for various estimation methods (minimum Kullback–Leibler, minimum Hellinger, and minimum L1 distances) of $\left(c_{T},a_{T}\right).$

These observations motivate the development of an enhanced estimation strategy.

### 5.2. An Enhanced Sequential Criterion for Estimation

To overcome the limitations of standard estimation methods, we introduce an additional estimation criterion that exploits the iterative structure of the procedure. The main idea is to enforce stability by ensuring coherence between successive conditional quantile estimates through a backward validation mechanism.

Due to its backwards nature, this estimation procedure stands for iterations j=2,…,m.

At iteration j−1, the sample $Y_{j-1,i}$, 1≤i≤K is collected in the form of binary responses

$$Y_{j-1,i}=1_{{\tilde{R}}_{j-1,i}\geq {\tilde{s}}_{j-1}}.$$

where ${\tilde{R}}_{j-1,i}$ are drawn from $G_{{\left(c,a\right)}_{j-2}\;}$. The empirical failure probability given by

(8) $${\hat{p}}_{j-1}:=\frac{1}{K}\sum_{i=1}^{n}Y_{j-1,i}.$$

provides an estimate of $P\left(\tilde{R}>{\tilde{s}}_{j-1}|\tilde{R}>{\tilde{s}}_{j-2}\right)$ and a proxy of *p*. The conditional probability $P\left(\tilde{R}>{\tilde{s}}_{j-1}|\tilde{R}>{\tilde{s}}_{j-2}\right)$ can be written as a function of the estimated parameters obtained at iteration *j*, namely ${\left(\hat{c},\hat{a}\right)}_{j}$ using (7). This relies on the stability of the GPD under threshold exceedances, which ensures that parameters estimated at level *j* remain consistent with the distribution at previous levels.

At step j, estimate $P\left(\tilde{R}>{\tilde{s}}_{j-1}|\tilde{R}>{\tilde{s}}_{j-2}\right)$ making use of $G_{{\left(\hat{c},\hat{a}\right)}_{j}}.$ This backward estimator writes

$$\frac{{\bar{G}}_{{\left(\hat{c},\hat{a}\right)}_{j}}\left({\tilde{s}}_{j-1}\right)}{{\bar{G}}_{{\left(\hat{c},\hat{a}\right)}_{j}}\left({\tilde{s}}_{j-2}\right)}=1-G_{{\left(\hat{c},\hat{a}\right)}_{j}}\left({\tilde{s}}_{j-1}-{\tilde{s}}_{j-2}\right).$$

The distance

(9) $$\left|\left({\bar{G}}_{{\left(\hat{c},\hat{a}\right)}_{j}}\left({\tilde{s}}_{j-1}-{\tilde{s}}_{j-2}\right)\right)-\hat{p_{j-1}}\right|$$

should be small, since both ${\bar{G}}_{{\left(\hat{c},\hat{a}\right)}_{j}}\left({\tilde{s}}_{j-1}-{\tilde{s}}_{j-2}\right)$ and $\;{\hat{p}}_{j-1}$ should approximate p.

Consider the distance between quantiles

(10) $$\left|\left({\tilde{s}}_{j-1}-{\tilde{s}}_{j-2}\right)-G_{{\left(\hat{c},\hat{a}\right)}_{j}}^{-1}\left(1-{\hat{p}}_{j-1}\right)\right|.$$

An estimate ${\left(\hat{c},\hat{a}\right)}_{j}$ can be proposed as the minimizer of the above expression for $\left({\tilde{s}}_{j-1}-{\tilde{s}}_{j-2}\right)$ for all *j*. This backward estimation provides coherence with respect to the unknown initial distribution $G_{\left(c_{T},a_{T}\right)}$ through a retroactive validation of the parameters. If we had started with a good guess $\left(\hat{c},\hat{a}\right)=\left(c_{T},a_{T}\right)$, then the successive ${\left(\hat{c},\hat{a}\right)}_{j},\;{\tilde{s}}_{j-1}$, etc., would make (10) small, since ${\tilde{s}}_{j-1}$ (resp. ${\tilde{s}}_{j-2}$) would estimate the p-conditional quantile of $P\left(\left..\right|\tilde{R}>{\tilde{s}}_{j-2}\right)$ (resp. $P\left(\left..\right|\tilde{R}>{\tilde{s}}_{j-3}\right)$).

It remains to restrict the minimization of (10) to a set of plausible parameter values. We suggest constructing a confidence region for the parameter $\left(c_{T},a_{T}\right).$ With ${\hat{p}}_{j}$ defined in (8) and $\gamma \in \left(0,1\right)$, define the (1−γ)-confidence region for *p* by

(11) $$I_{1-\gamma }=\left[{\hat{p}}_{j}-z_{1-\gamma /2}\sqrt{\frac{{\hat{p}}_{j}\left(1-{\hat{p}}_{j}\right)}{K-1}};{\hat{p}}_{j}+z_{1-\gamma /2}\sqrt{\frac{{\hat{p}}_{j}\left(1-{\hat{p}}_{j}\right)}{K-1}}\right]$$

where $z_{\tau }$ is the τ-quantile of the standard normal distribution. Define

(12) $$S_{j}=\left\{\left(c,a\right):\left(1-G_{\left(c,a\right)}\left({\tilde{s}}_{j}-{\tilde{s}}_{j-1}\right)\right)\in I_{1-\gamma }\right\}.$$

Therefore, $S_{j}$ is a plausible set for $({\hat{c}}_{T},{\hat{a}}_{T}).$

We summarize this discussion:

At iteration j, the estimator of $\left(c_{T},a_{T}\right)$ is a solution of the minimization problem

(13) $$\min_{\left(c,a\right)\in S_{j}}\left|\left({\tilde{s}}_{j-1}-{\tilde{s}}_{j-2}\right)-G_{\left(c,a+c{\tilde{s}}_{j-2}\right)}^{-1}\left(1-{\hat{p}}_{j-1}\right)\right|.$$

where optimization is performed using standard numerical methods.

This backward estimation procedure enforces coherence with respect to the unknown initial distribution $G_{\left(c_{T},a_{T}\right)}$ by ensuring consistency between successive conditional quantile estimates. If the initial parameters were equal to the true values $\left(c_{T},a_{T}\right)$, then the successive thresholds ${\tilde{s}}_{j}$ would accurately track the corresponding conditional quantiles, making the discrepancy in (10) negligible.

This criterion can be interpreted as a backward validation mechanism, in the spirit of cross-validation or jackknife-type approaches, where estimates at a given stage are assessed through their ability to reproduce quantities observed at previous stages. From a statistical perspective, it reflects the sequential nature of the data acquisition process by incorporating past information into the current estimation step, thereby ensuring coherence between successive conditional quantile estimates. It should therefore not be viewed as a likelihood-based or regularization approach, but rather as a consistency-based validation criterion embedded within the sequential design.

### 5.3. Theoretical Insight into the Estimator

Although a full theoretical analysis of the proposed estimator is beyond the scope of this paper, some insight into its behavior can be provided. The method relies on a splitting strategy that decomposes a rare event into a sequence of conditional events, thereby progressively exploring the tail of the distribution. Each step involves the estimation of an intermediate conditional quantile, and the final extreme quantile is obtained by the composition of these levels.

From an extreme value theory perspective, this construction can be interpreted as iteratively approximating increasingly extreme quantiles of the original distribution. As the number of iterations increases, the procedure effectively focuses on the tail region, where classical asymptotic results for conditional excess distributions apply.

Moreover, the recursive structure of the algorithm contributes to its stability: provided that the parameter estimates at a given step are sufficiently accurate, the subsequent sampling is concentrated in a relevant region of the distribution. The enhanced estimation criterion introduced in Section 5 further reinforces this stability by promoting consistency between successive conditional quantile estimates. From a heuristic perspective, the sequential updating mechanism may be interpreted as a fixed-point-type iteration, where parameter estimates are adjusted to ensure consistency with previously observed conditional quantiles. This suggests that convergence may be expected under suitable conditions, although no formal result is established in the present work. This reasoning relies on asymptotic arguments from extreme value theory, in particular on the applicability of the De Haan theorem, which ensures that the tail behavior can be approximated by a generalized Pareto distribution. Overall, these elements provide intuition on the expected convergence behavior of the method, even though a formal proof of consistency remains an open question.

### 5.4. Simulation-Based Numerical Results

This procedure has been applied in three cases. The reference case is $\left(c_{T},a_{T}\right)=\left(1.5,1.5\right)$. The second case, $\left(c_{T},a_{T}\right)=\left(0.8,1.5\right)$, corresponds to a lighter tail than the reference. The third case, $\left(c_{T},a_{T}\right)=\left(1.5,3\right)$, corresponds to a distribution with the same tail index as the reference but a larger dispersion index.

The overall procedure can be summarized as follows in Procedure 2:

Performance is primarily assessed using relative error, which provides a scale-invariant measure particularly suited to extreme quantile estimation, where the quantities of interest may span several orders of magnitude. From a practical standpoint, this metric also reflects the ability of the method to recover the correct order of magnitude, which is often the main objective in industrial applications.

Table 6 highlights important features of the proposed estimator. First, the estimation of ${\tilde{q}}_{1-\alpha }$ deteriorates as the tail of the distribution becomes heavier, reflecting the intrinsic difficulty of extrapolating further into the tail. In addition, the estimator tends to underestimate ${\tilde{q}}_{1-\alpha }$, implying a conservative nature of the sequential exploration of extreme regions.

**Figure 4:**
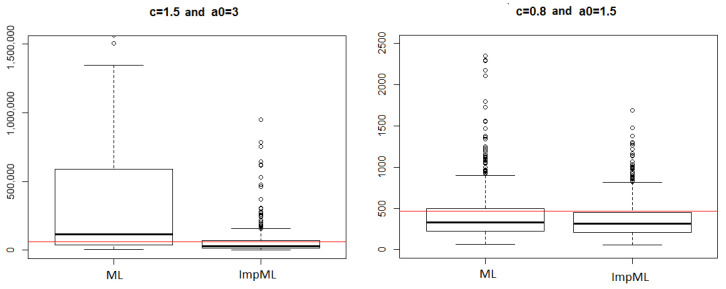
Estimations of the (1−α)-quantile of two GPD obtained by Maximum Likelihood and by the improved Maximum Likelihood method. The red line stands for the real value of $q_{\alpha }$.

Despite these limitations, the proposed method shows a clear improvement over the simple Maximum Likelihood estimation. This gain is particularly pronounced in the case of heavy-tailed distributions, where classical approaches struggle to provide reliable estimates Figure 4.
**Procedure 2** Simulation and dual-criterion estimation procedure**Initialization:**Fix the design parameters *p*, *m*, and *K*. In the following examples, we set m=5, the target level p=0.25, and K∈{15,30,50}.Select an initial threshold ${\tilde{s}}_{1}$ based on expert knowledge and perform *K* trials at this level to obtain a first estimate of $\left(c_{T},a_{T}\right)$. Since ${\tilde{s}}_{1}$ is not calibrated to exactly match the target level *p*, the resulting probability $p_{1}$ may differ from *p*. Therefore, *p* is adjusted using ${\hat{p}}_{1}$ so as to satisfy ${\hat{p}}_{1}p^{m-1}\approx \alpha$.**For each stage j=2,…,m:****1. Generation of *K* trials:**Perform *K* trials at the current threshold ${\tilde{s}}_{j}$, leading to binary observations$$Y_{j,i}=1_{{\tilde{R}}_{j,i}\geq {\tilde{s}}_{j}},\;i=1,\ldots ,K,$$
where ${\tilde{R}}_{j,i}$ are simulated under $G_{{\left(c,a\right)}_{j-1}}$.Compute the empirical conditional probability$${\hat{p}}_{j}=\frac{1}{K}\sum_{i=1}^{K}Y_{j,i},$$
and construct a confidence interval $I_{1-\gamma }\left({\hat{p}}_{j}\right)$ with confidence level 1−γ=0.8 (see Section 5.2).**2. Parameter space restriction:**Define the set of admissible parameters $S_{j}$ as those for which the theoretical conditional probability under the Generalized Pareto model lies within the confidence interval $I_{1-\gamma }$ (see (11) and (12)).**3. Stability criterion:**Among the candidates in $S_{j}$, select the parameter pair ${\left(\hat{c},\hat{a}\right)}_{j}$ by minimizing the discrepancy between successive conditional quantile estimates:$${\left(\hat{c},\hat{a}\right)}_{j}=\arg\min_{\left(c,a\right)\in S_{j}}\left|\left({\tilde{s}}_{j-1}-{\tilde{s}}_{j-2}\right)-G_{\left(c,a+c{\tilde{s}}_{j-2}\right)}^{-1}\left(1-{\hat{p}}_{j-1}\right)\right|.$$From the estimated parameters, retrieve the (1−p)-quantile of the estimated conditional distribution $G_{{\left(\hat{c},\hat{a}\right)}_{j}}$:$${\tilde{s}}_{j+1}=G_{{\left(\hat{c},\hat{a}\right)}_{j}}^{-1}\left(1-p\right)+{\tilde{s}}_{j},$$**4. Final estimator:**The estimator of the target extreme quantile ${\tilde{q}}_{1-\alpha }$ is given by the final threshold ${\tilde{s}}_{m}$.

**Figure 5:**
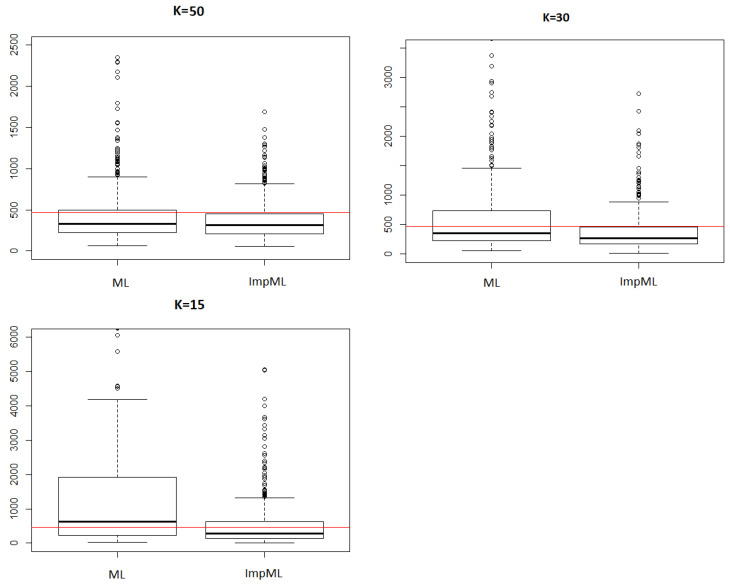
Estimations of the (1−α)-quantile of a GPD(0.8,1.5) obtained by Maximum Likelihood and by the improved Maximum Likelihood method for different values of *K*. The red line stands for the real value of $q_{\alpha }$.

Moreover, the influence of the number *K* of replications at each step reveals an important advantage of the method. While reducing *K* naturally increases variability, it also amplifies the relative gain over Maximum Likelihood estimation. This behavior, illustrated in Figure 5, suggests a certain robustness of the proposed approach in low-information settings, where only a limited number of trials can be performed at each stage.

### 5.5. Performance of the Sequential Estimation

As stated in Section 5, a substantial amount of information is lost under complete truncation and binary sampling.

As stated in Section 3, most available approaches either assume fully observed data or rely on strong prior knowledge of the underlying distribution, particularly in the tail region. These assumptions are not compatible with the binary observation framework considered here. Thus, direct comparisons with existing methods for extreme quantile estimation are not straightforward in our setting.

For this reason, we instead compare our procedure to a benchmark based on full data observations, which provides an upper bound on the achievable performance under ideal information conditions.

#### 5.5.1. Estimation of an Extreme Quantile Based on Complete Data, De Valk’s Estimator

In order to provide an upper bound for performance, we use the estimator proposed by De Valk and Cai (2018) [6]. Their framework targets quantiles of order $p_{n}\in \left[n^{-\tau_{1}},\;n^{-\tau_{2}}\right]$ with $\tau_{2}>\tau_{1}>1$, where *n* is the sample size, which aligns with the industrial context that motivated this work. De Valk’s proposal is a modified Hill estimator adapted to log-Weibull-tailed models. De Valk’s estimator is consistent, asymptotically normally distributed, though it may exhibit finite-sample bias.

We briefly recall the assumptions underpinning De Valk’s approach. Let $X_{1},\ldots ,X_{n}$ be *n* iid r.v’s with distribution *F*, and denote $X_{k:n}$ the k− order statistics. A tail regularity assumption is needed in order to estimate a quantile with order greater than 1−1/n.

Denote $U\left(t\right)=F^{-1}\left(1-1/t\right)$, and let the function *q* be defined by

$$q\left(y\right)=U\left(e^{y}\right)=F^{-1}\left(1-e^{-y}\right)$$

for y>0.

Assume that

(14) $$\lim_{y\to \infty }\;\frac{\log q(y\lambda )-\log q(y)}{g(y)}=h_{\theta }\left(\lambda \right)\;\;\;\lambda >0$$

where *g* is a regularly varying function and

$$h_{\theta }\left(\lambda \right)=\left\{\begin{array}{c} \frac{\lambda^{\theta }-1}{\theta }\;\mathrm{if}\;\theta \neq 0\\ \log\lambda \;\mathrm{if}\;\theta =0 \end{array}\right.$$

de Valk writes condition (14) as $\log q\in ERV_{\theta }\left(g\right)$.

Despite its naming of log-Generalized tails, this condition also holds for Pareto-tailed distributions, as can be checked, providing θ=1.

We now introduce de Valk’s extreme quantile estimator.

Let

$$\vartheta_{k,n}:=\sum_{j=k}^{n}\frac{1}{j}.$$

Let q(z) be the quantile of order $e^{-z}=p_{n}$ of the distribution *F*. The estimator makes use of $X_{n-l_{n}:n}$, an intermediate order statistic of $X_{1},\ldots ,X_{n}$, where $l_{n}$ tends to infinity as n→∞ and $l_{n}$/n→0.

de Valk’s estimator writes

(15) $$\hat{q}\left(z\right)=X_{n-l_{n}:n}\exp\left\{g\left(\vartheta_{l_{n},n}\right)h_{\theta }\left(\frac{z}{\vartheta_{l_{n+1},n}}\right)\right\}.$$

When the support of *F* overlaps $R^{-}$, then the sample size *n* should be large; see de Valk [6] for details.

Note that, in the case of a GPD(c,a), parameter θ is known and equal to 1, and the normalizing function *g* is defined by g(x)=cx for x>0.

#### 5.5.2. Loss in Accuracy Due to Binary Sampling

Table 7 compares the performance of De Valk’s method (using complete data) with that of our sequential procedure (using only dichotomous outcomes). Unsurprisingly, de Valk’s estimator outperforms ours. This gap reflects the loss of information induced by thresholding and dichotomization. Nevertheless, the comparison remains informative: even though our estimator typically exhibits a larger bias, its dispersion is of the same order of magnitude when handling heavy-tailed GPD models. Given the binary nature of the data, the average relative error is quite honorable. Overall, we can assess that a large part of the volatility of the estimator produced by our sequential methodology is due to the nature of the GPD model, as well as to the sample size.

## 6. Sequential Design for the Weibull Model

The main property that led to the GPD model is its relevance for tail modeling and its stability through threshold conditioning. In this section, we also investigate a parameterization based on the Weibull distribution as one of the most classical and widely used models in reliability engineering. Under this assumption, the conditional distribution of $\tilde{R}$ given $\left\{\tilde{R}>s\right\}$ takes a rather simple form, which allows for some variation of the sequential design method.

### 6.1. The Weibull Model

Let $\tilde{R}∼\mathrm{Weibull}\left(\alpha ,\beta \right)$ with scale α>0 and shape β>0, and denote by *G* its c.d.f., *g* its density, and $G^{-1}$ the quantile function. For x≥0,

$$\begin{array}{cc} \;G(x) & =1-\exp\left(-{\left(\frac{x}{\alpha }\right)}^{\beta }\right)\\ \;\mathrm{for}\;0<u<1,\;\;G^{-1}\left(u\right) & =\alpha {\left(-\log\left(1-u\right)\right)}^{1/\beta } \end{array}$$

The conditional distribution of $\tilde{R}$ given truncation above a threshold is a truncated Weibull.

$$\begin{array}{cc} \;\mathrm{for}\;{\tilde{s}}_{2}>{\tilde{s}}_{1},\;\;P\left(\tilde{R}>{\tilde{s}}_{2}|\tilde{R}>{\tilde{s}}_{1}\right) & =\frac{P(\tilde{R}>{\tilde{s}}_{2})}{P(\tilde{R}>{\tilde{s}}_{1})}\\ & =\exp\left\{\left(-{\left(\frac{s_{2}}{\alpha }\right)}^{\beta }+{\left(\frac{s_{1}}{\alpha }\right)}^{\beta }\right)\right\} \end{array}$$

Let $G_{s_{2}}$ denote the distribution function of $\tilde{R}$ given $\left(\tilde{R}>{\tilde{s}}_{2}\right)$.

A useful identity follows. For ${\tilde{s}}_{2}>{\tilde{s}}_{1}$,

(16) $$\log P\left(\tilde{R}>{\tilde{s}}_{2}|\tilde{R}>{\tilde{s}}_{1}\right)=\left[{\left(\frac{{\tilde{s}}_{2}}{{\tilde{s}}_{1}}\right)}^{\beta }-1\right]\log P\left(\tilde{R}>{\tilde{s}}_{1}\right)$$

Assuming $P(\tilde{R}>{\tilde{s}}_{1})=p$ and given ${\tilde{s}}_{1}$, we may find ${\tilde{s}}_{2}$ the conditional quantile of order 1−p of the distribution of $\tilde{R}$ given $\left\{\tilde{R}>{\tilde{s}}_{1}\right\}$. This solves the first step of the sequential estimation procedure through

$$\log p=\left[{\left(\frac{{\tilde{s}}_{2}}{{\tilde{s}}_{1}}\right)}^{\beta }-1\right]\log p$$

where the parameter β has to be estimated on the first run of trials.

The same logic extends iteratively. For ${\tilde{s}}_{j+1}>{\tilde{s}}_{j}>{\tilde{s}}_{j-1}$

(17) $$\begin{array}{cc} \log P(\tilde{R}>{\tilde{s}}_{j+1}|\tilde{R}>{\tilde{s}}_{j}) & =\left[\frac{\log P(\tilde{R}>{\tilde{s}}_{j+1}|\tilde{R}>{\tilde{s}}_{j-1})}{\log P(\tilde{R}>{\tilde{s}}_{j}|\tilde{R}>{\tilde{s}}_{j-1})}-1\right]\log P\left(\tilde{R}>{\tilde{s}}_{j}|\tilde{R}>{\tilde{s}}_{j-1}\right)\\ \; & =\left[\frac{{\tilde{s}}_{j-1}^{\beta }-{\tilde{s}}_{j+1}^{\beta }}{{\tilde{s}}_{j-1}^{\beta }-{\tilde{s}}_{j}^{\beta }}-1\right]\log P\left(\tilde{R}>{\tilde{s}}_{j}|\tilde{R}>{\tilde{s}}_{j-1}\right) \end{array}$$

At iteration *j*, the thresholds ${\tilde{s}}_{j}$ and ${\tilde{s}}_{j-1}$ are known; the threshold ${\tilde{s}}_{j+1}$ is the (1−p)-quantile of the conditional distribution, $P(\tilde{R}>{\tilde{s}}_{j+1}|\tilde{R}>{\tilde{s}}_{j})=p$, hence solving

$$\log p=\left[\frac{{\tilde{s}}_{j-1}^{\beta }-{\tilde{s}}_{j+1}^{\beta }}{{\tilde{s}}_{j-1}^{\beta }-{\tilde{s}}_{j}^{\beta }}-1\right]\log p$$

where the estimate of β is updated from the data collected at iteration j.

### 6.2. Numerical Results

In line with Section 5.4 and Section 5.5, we assess the performance of the sequential design under a Weibull model. We estimate the (1−α)-quantile in three scenarios. In the first case, the scale parameter *a* and the shape parameter *b* satisfy $\left(a,b\right)=\left(3,0.9\right)$. This corresponds to a strictly decreasing density function with a heavy tail. In the second case, (a,b)=(3,1.5), the distribution is more skewed; in the third, (a,b)=(2,1.5), the distribution is less dispersed with a lighter tail.

Table 8 shows that the performance of our procedure is, as expected, sensitive to the shape of the distribution. The estimators are less accurate in case 1, corresponding to a heavier tail. We compare these errors to those obtained with complete data using de Valk’s methodology. The loss of accuracy due to data deterioration (binary sampling and truncation) is similar to that observed in the Pareto case, though slightly more pronounced. This is consistent with the fact that the Weibull family is less naturally aligned with the splitting structure than the GPD, which enjoys exact stability under threshold conditioning.

## 7. Model Selection and Misspecification

In the above sections, we considered two models primarily motivated by their theoretical properties. As stated in Section 4.3, modeling $\tilde{R}$ by a GPD(c,a) with c>0 is justified when the support of the original variable *R* may be bounded by 0. Note, however, that the GPD model naturally extends to c=0, which corresponds to the exponential distribution and represents a trivial limiting case for excess probabilities.

Although we excluded the exponential case when modeling the excess probabilities of $\tilde{R}$ with a GPD, we nevertheless considered the Weibull model in Section 6, which lies in the maximum domain of attraction associated with c=0. Beyond its compatibility with the splitting structure, the Weibull law is a classical and widely used model in reliability, which makes it a natural candidate for an adapted version of our sequential method.

In this section, we discuss the modeling decisions and give some hints on how to deal with misspecification.

### 7.1. Model Selection

The decision between a Pareto-type model with a strictly positive tail index and a Weibull-type model has been addressed in the literature; a variety of tests exist for assessing membership to a given maximum domain of attraction.

Dietrich et al. (2002) [19] Drees et al. (2006) [20] both propose a test for extreme value conditions related to Cramer-von Mises tests. Let *X* of distribution function *G*. The null hypothesis is

$$H_{0}:G\in MDA\left(c_{0}\right).$$

In our case, the theoretical value for the tail index is $c_{0}=0$. The former test builds on the tail empirical quantile function, while the latter relies on a weighted approximation of the tail empirical distribution.

Choulakian and Stephens (2001) [21] propose a goodness-of-fit test in the spirit of Cramér–von Mises statistics, where unknown parameters are replaced by maximum likelihood estimates. Given a sample $X_{1},\ldots ,X_{n}$ from *G*, the hypothesis to be tested is $H_{0}$: the sample stems from a $GPD(c_{0},\hat{a})$. The associated test statistics are given by the following equation:

$$\begin{array}{cc} & W^{2}=\sum_{i=1}^{n}{\left(\hat{G}\left(x_{\left(i\right)}\right)-\frac{2i-1}{2n}\right)}^{2}+\frac{1}{12n};\\ & A^{2}=-n-\frac{1}{n}\sum_{i=1}^{n}\left(2i-1\right)\left\{\log\left(\hat{G}\left(x_{\left(i\right)}\right)\right)+\log\left(1-\hat{G}\left(x_{(}n+1-i\right)\right)\right\}, \end{array}$$

where $x_{\left(i\right)}$ denotes the i-th order statistic of the sample. The authors provide the corresponding tables of critical points.

Jurečková and Picek (2001) [22] designed a non-parametric test for determining whether a distribution *G* is light or heavy-tailed. The null hypothesis is defined by the following:

$$H_{c_{O}}:x^{1/c_{0}}\left(1-G\left(x\right)\right)\leq 1\;\;\forall x>x_{0}\;\mathrm{for}\;\mathrm{some}\;x_{0}>0$$

with fixed hypothetical $c_{0}$. The test procedure consists of splitting the data set into *N* samples and computing the empirical distribution of the extrema of each sample.

The practical assessment of model suitability in our industrial context is delicate. Two main obstacles arise: (i) data are collected sequentially during the procedure, with no upfront sample of available observations; (ii) the variable of interest *R* is not observed directly and only dichotomized exceedances over chosen thresholds are available. Most existing procedures assume fully observed data and are semi- or nonparametric tests built from order statistics; their performance also relies on large samples, which are unrealistic in our setting due to the cost and duration of experimental trials.

As an alternative, one may resort to *a posteriori* validation, once the procedure is completed, combining the statistical output with expert judgment to confirm (or reject) the chosen model.

### 7.2. Misspecification

In Section 4.3, we assumed that $\tilde{R}$ follows a GPD at the outset. In practice, the tail of the true distribution may converge toward a GPD as thresholds increase, yet differ from it at finite levels.

#### 7.2.1. Robustness to Model Misspecification

The proposed approach relies on parametric assumptions, in particular the generalized Pareto distribution, which is motivated by its stability under threshold exceedances in extreme value theory. This choice is especially relevant in the later stages of the splitting procedure, where the algorithm focuses on increasingly extreme regions of the distribution.

It should be noted, however, that the parametric assumption may be less accurate during the early iterations, when the data are not yet in the tail regime. Nevertheless, the sequential nature of the method progressively reduces the influence of these initial stages, as the final estimate is primarily driven by the deepest levels of the splitting procedure.

This behavior is consistent with standard extreme value estimation techniques, which rely primarily on the largest observations and are therefore relatively insensitive to the distribution in central regions. As a result, the method is expected to exhibit some degree of robustness to model misspecification, provided that the tail behavior is adequately captured.

A more systematic assessment of this robustness, for instance, through simulation studies under alternative distributions, would be a valuable direction for future work. However, such an analysis is not straightforward in the present sequential setting, where data are generated adaptively under conditional distributions. In particular, standard tools such as influence functions are difficult to implement, and a comprehensive empirical validation would go beyond the scope of the present work.

#### 7.2.2. Insights on Control of Model Deviation

While the previous subsection discusses the robustness of the proposed approach from an asymptotic perspective, it is also important to quantify the effect of model misspecification in finite-sample settings, especially in the early iterations of the procedure, where the extreme regime has not yet been reached.

In this subsection, we address this issue by introducing a neighborhood-based framework that allows us to control deviations from the generalized Pareto model and to guide the selection of appropriate threshold levels within the sequential design.

Let us assume that $\tilde{R}$ does not follow a GPD with distribution function *F*, but rather a distribution *G* whose tail becomes increasingly close to that of a GPD.

In this case, the issue is to control the distance between *G* and the theoretical GPD and to determine a threshold level beyond which this discrepancy becomes negligible. One way to proceed is to restrict attention to a neighborhood of *F*:

(18) $$V_{\epsilon }\left(F\right)=\left\{G:\underset{x}{\sup}\left|\bar{F}\left(x\right)-\bar{G}\left(x\right)\right|w\left(x\right)\leq \epsilon \right\},$$

where ϵ≥0 and *w* is an increasing weight function such that $\lim_{x\to \infty }w\left(x\right)=\infty$. $V_{\epsilon }\left(F\right)$ defines a neighborhood, which does not tolerate large departures from *F* in the right tail of the distribution.

Let x≥s. It follows from (18) that a bound for the conditional distribution of *x* given R>s is as follows:

(19) $$\frac{\bar{F}\left(x\right)-\epsilon /w\left(x\right)}{\bar{F}\left(s\right)+\epsilon /w\left(s\right)}\leq \frac{\bar{G}\left(x\right)}{\bar{G}\left(s\right)}\leq \frac{\bar{F}\left(x+\right)+\epsilon /w\left(x\right)}{\bar{F}\left(s\right)-\epsilon /w\left(s\right)}.$$

When ϵ=0, the bounds of (19) match the conditional probabilities of the theoretical Pareto distribution.

In order to control the distance between *F* and *G*, this bound may be rewritten in terms of relative error with respect to the Pareto distribution. Using a first-order Taylor expansion for small ϵ, we obtain

(20) $$1-u\left(s,x\right).\epsilon \leq \frac{\frac{\bar{G}\left(x\right)}{\bar{G}\left(s\right)}}{\frac{\bar{F}\left(x\right)}{\bar{F}\left(s\right)}}\leq 1+u\left(x,s\right).\epsilon ,$$

where

$$u\left(s,x\right)=\frac{{\left(1+\frac{cs}{a}\right)}^{1/c}}{w(s)}+\frac{{\left(1+\frac{cx}{a}\right)}^{1/c}}{w(x)}.$$

For a given ϵ close to 0, the relative error on the conditional probabilities can be controlled through the choice of *s*. In particular, the relative error is bounded by a prescribed level δ>0 whenever

$$\frac{{\left(1+\frac{cs}{a}\right)}^{1/c}}{w(s)}\leq \frac{\delta }{\epsilon }\frac{{\left(1+\frac{cx}{a}\right)}^{1/c}}{w(x)}.$$

This provides a practical guideline for selecting thresholds that mitigate the impact of misspecification within the sequential design.

## 8. Perspectives, Generalization of the Two Models

In this work, we have considered two models for $\tilde{R}$ that exploit the thresholding operations used in the splitting method. This choice is, however, restrictive: the limited information obtained from the trials prevents the use of highly flexible models for the distribution of the resistance. In the following, we outline several possible extensions and generalizations of those models based on common properties of the GPD and Weibull models.

### 8.1. Variations Around Mixture Forms

When the tail index is positive, the GPD is completely monotone, and thus can be written as the Laplace transform of a probability distribution. Thyrion (1964) [23] and Thorin (1977) [24] established that a $GPD(a_{T},c_{T})$, with $c_{T}>0$, can be written as the Laplace transform of a Gamma random variable *V* whose parameters are functions of $a_{T}$ and $c_{T}$: $V\;∼\;\Gamma \left(\frac{1}{c_{T}},\frac{a_{T}}{c_{T}}\right)$. Let *v* denote the density of *V*,

(21) $$\begin{array}{cc} \forall x\geq 0,\;\;\bar{G}\left(x\right)= & \int_{0}^{\infty }\exp\left(-xy\right)v\left(y\right)dy\\ \; & \;\mathrm{where}\;\;\;v\left(y\right)=\frac{{\left(a_{T}/c_{T}\right)}^{1/c}}{\Gamma (1/c_{T})}y^{1/c_{T}-1}\exp\left(-\frac{a_{T}y}{c_{T}}\right). \end{array}$$

It follows that the conditional survival function of $\tilde{R}$, ${\bar{G}}_{s_{j}}$, is given by the following:

$$\begin{array}{cc} P(\tilde{R}>{\tilde{s}}_{j+1}|{\tilde{R}}_{j}>{\tilde{s}}_{j}) & ={\bar{G}}_{{\tilde{s}}_{j}}\left({\tilde{s}}_{j+1}-{\tilde{s}}_{j}\right)\\ & =\int_{0}^{\infty }\exp\left\{-({\tilde{s}}_{j+1}-{\tilde{s}}_{j})y\right\}v_{j}\left(y\right)dy,\\ & \;\;\mathrm{where}\;V_{j}\;\mathrm{is}\;a\;\mathrm{random}\;\mathrm{variable}\;\mathrm{of}\;\mathrm{distribution}\;\Gamma \left(\frac{1}{c_{j}},\frac{a_{j}}{c_{j}}\right). \end{array}$$

with $c_{j}=c_{T}$ and $a_{j}=a_{j-1}+c_{T}\left({\tilde{s}}_{j}-{\tilde{s}}_{j-1}\right)$.

Expression (21) gives room to an extension of the Pareto model. Indeed, we could consider distributions of $\tilde{R}$ that share the same mixture form with a mixing variable *W* that possesses some common characteristics with the Gamma-distributed random variable V.

Similarly, the Weibull distribution W(α,β) can also be written as the Laplace transform of a stable law of density *g* whenever β≤1. Indeed, it holds from Feller 1971 [25] (p. 450, Theorem 1) that:

(22) $$\forall x\geq 0,\;\;\exp\left\{-x^{\beta }\right\}=\int_{0}^{\infty }\exp\left(-xy\right)g\left(y\right)dy$$

where *g* is the density of an infinitely divisible probability distribution.

It follows that, for $s_{j}<s_{j+1}$

(23) $$\begin{array}{cc} P(\tilde{R}>{\tilde{s}}_{j+1}|{\tilde{R}}_{j}>{\tilde{s}}_{j}) & =\frac{\exp\left\{-{\left({\tilde{s}}_{j+1}/\alpha \right)}^{\beta }\right\}}{\exp\left\{-{\left({\tilde{s}}_{j}/\alpha \right)}^{\beta }\right\}}\\ \; & =\frac{\int_{0}^{\infty }\exp\left\{(-\left({\tilde{s}}_{j+1}/\alpha \right)y\right\}g\left(y\right)dy}{\int_{0}^{\infty }\exp\left\{-({\tilde{s}}_{j}/\alpha )y\right\}g\left(y\right)dy}=\frac{\int_{0}^{\infty }\exp\left\{-({\tilde{s}}_{j+1}/\alpha )y\right\}g\left(y\right)dy}{K(s_{j})}\\ \; & =\frac{1}{K(s_{j})}\int_{0}^{\infty }\exp\left\{-{\tilde{s}}_{j+1}u\right\}g_{\alpha }\left(u\right))du\\ \; & \;\;\;\mathrm{with}\;u=y/\alpha \;\mathrm{and}\;g_{\alpha }\left(u\right)=\alpha g\left(\alpha u\right) \end{array}$$

Thus, an alternative modeling of $\tilde{R}$ could consist of any distribution that can be written as a Laplace transform of a stable law of density $w_{\alpha ,\beta }$ defined on $R_{+}$ and parametrized by (α,β), that satisfies the following property. For any s>0, the distribution function of the conditional distribution of $\tilde{R}$ given $\tilde{R}>s$ can be expressed as the Laplace transform of $w_{\alpha ,\beta }^{\left(\alpha ,s\right)}\left(.\right)$ where

$$x>s,w_{\alpha ,\beta }^{\left(\alpha ,s\right)}\left(x\right)=\frac{\alpha w_{\alpha ,\beta }\left(\alpha x\right)}{K(s)},$$

where K(.) is defined in (23).

### 8.2. Variation Around the GPD

Another approach, inspired by Naveau et al. (2016) [26], is to modify the model so that the distribution of $\tilde{R}$ converges to a GPD in the upper tail while taking a more flexible form near 0.

$\tilde{R}$ is generated through $G_{\left(c_{T},a_{T}\right)}^{-1}\left(U\right)$ with U∼U[0,1]. Let us consider now a deformation of the uniform variable $V=L^{-1}\left(U\right)$ defined on [0,1], and the transform *W* of the GPD: $W^{-1}\left(U\right)=G_{\left(c_{T},a_{T}\right)}^{-1}\left(L^{-1}\left(U\right)\right)$.

Since the survival function of the GPD is completely monotone, we can choose *W* so that the distribution of $\tilde{R}$ retains this property.

**Proposition** **2.**  *If ϕ:[0,∞[→R is completely monotone and ψ is a positive function whose derivative is completely monotone, then ϕ(ψ) is completely monotone.*

The transformation of the GPD has a cumulative distribution function $W=L(G(c_{T},a_{T}))$ and survival function $\bar{W}=\bar{L}\left(G(c_{T},a_{T}\right))$. $G(c_{T},a_{T})$ is a Bernstein function; thus, $\bar{W}$ is completely monotone if $\bar{L}$ is as well.

#### Examples of Admissible Functions

*(1) Exponential form:*

$$\begin{array}{cc} & L(0)=0\\ & L\left(x\right)=\frac{1-\exp(-\lambda x^{\alpha })}{1-\exp(-\lambda )}\;\;\;\mathrm{avec}\;0\leq \alpha \leq 1\;\mathrm{et}\;\lambda >0\\ & L(1)=1 \end{array}$$

The obtained transformation is: ∀x>0,

$${\bar{W}}_{\left(\lambda ,c_{T},a_{T}\right)}\left(x\right)=\bar{L}\left(G\left(x\right)\right)=\frac{\exp\left(-\lambda {\left[1-{\left(1+\frac{c_{T}}{a_{T}}\right)}^{-1/c_{T}}\right]}^{\alpha }\right)-\exp\left(-\lambda \right)}{1-\exp(-\lambda )}$$

with ${\bar{W}}_{\left(\lambda ,c_{T},a_{T}\right)}\left(x\right)$ completely monotone.

*(2) Logarithmic form:*

$$\begin{array}{cc} & L(0)=0\\ & L\left(x\right)=\frac{\log(x+1)}{\log2}\;\;\;\;\;(or\;more\;generally\frac{\log(\alpha x+1)}{\log2},\;\alpha >0)\\ & L(1)=1 \end{array}$$

and ∀x>0,

$${\bar{W}}_{\left(c_{T},a_{T}\right)}\left(x\right)=1-\frac{\log\left(2-{\left(1+\frac{c_{T}}{a_{T}}\right)}^{-1/c_{T}}\right)}{\log2}$$

*(3) Root form:*

$$\begin{array}{cc} & L(0)=0\\ & L\left(x\right)=\frac{\sqrt{x+1}-1}{\sqrt{2}-1}\\ & L(1)=1 \end{array}$$

and

$${\bar{W}}_{\left(c_{T},a_{T}\right)}x)=1-\frac{\sqrt{2-{\left(1+\frac{c_{T}x}{a_{T}}\right)}^{-1/c_{T}}}-1}{\sqrt{2}}$$

*(4) Fraction form:*

$$\begin{array}{cc} & L(0)=0\\ & L\left(x\right)=\frac{(\alpha +1)x}{x+\alpha },\;\;\alpha >0\\ & L(1)=1 \end{array}$$

and

$${\bar{W}}_{\left(\alpha ,c_{T},a_{T}\right)}\left(x\right)=1-\frac{\left(\alpha +1\right)\left(1-{\left(1+\frac{c_{T}x}{a_{T}}\right)}^{-1/c}\right)}{1-{\left(1+\frac{c_{T}x}{a_{T}}\right)}^{-1/c_{T}}+\alpha }$$

**Figure 6:**
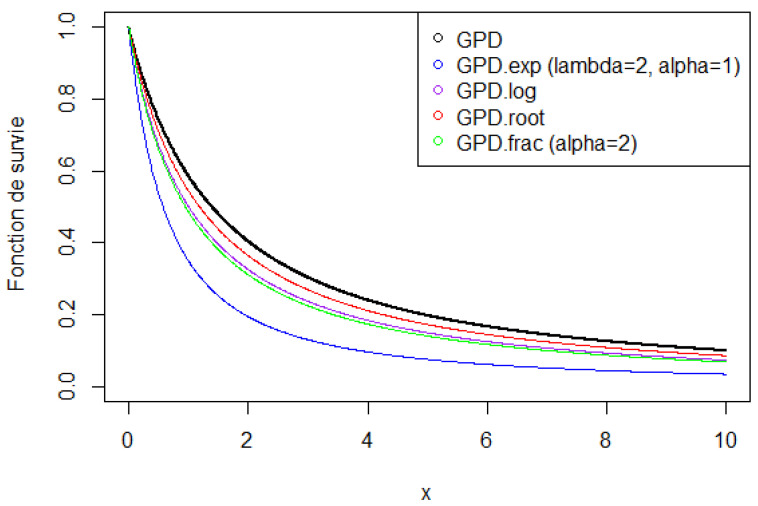
Survival functions associated with transformations of the GPD (0.8,1.5).

The shapes of the above transformations of the GPD are shown in Figure 6.

These transformations, however, do not preserve the stability under thresholding specific to the Pareto family; as a consequence, their implementation does not lead to a stable sequential procedure. Nonetheless, they illustrate how the proposed models can be generalized in contexts where additional information on the underlying variable is available.

## 9. Conclusions

The splitting-based procedure presented in this article proposes an innovative experimental design for estimating an extreme failure quantile. Its development is motivated, on the one hand, by major industrial stakes, and on the other, by the limited relevance of existing methodologies in this context. The core difficulty lies in the nature of the available information: the variable of interest is latent and only exceedance indicators over chosen thresholds can be observed.

We proposed a strategy based on splitting methods to decompose the target rare event into a sequence of more tractable conditional events. The splitting formula introduces a formal decomposition that we translated into a practical sampling strategy targeting the tail of the distribution of interest progressively.

The algebraic structure of the splitting equation motivated specific parametric assumptions on the underlying distribution. We considered two models that benefit from stability properties under thresholding: a Generalized Pareto Distribution (GPD) and a Weibull distribution. Building on this structure, we designed an estimation procedure that leverages the iterative nature of the design by combining a classical maximum likelihood criterion with a backward-consistency criterion on sequentially estimated conditional quantiles. The performance of the resulting estimator was assessed numerically. Although accuracy is necessarily constrained by the quantity and quality of information, the results can still be compared—at least in terms of order of magnitude—to what could be obtained under the idealized setting of fully observed data.

From a practical standpoint, while the GPD aligns most naturally with the splitting structure (due to its exact stability under threshold conditioning), the Weibull distribution remains highly relevant for reliability modeling and engineering practice.

The applicability of the proposed framework, however, depends on the ability to implement conditional sampling schemes, which may require a preliminary analysis of the system under study. While this may limit direct applicability in some contexts, it is worth noting that methods addressing similar problems typically rely on even stronger prior knowledge of the underlying distribution. In this respect, the present approach provides a relatively generic framework, requiring more limited prior information. A concrete illustration of such an implementation is provided in [1].

Another important aspect concerns the choice of the parametric model. The assumptions introduced to ensure tractability of the sequential design are motivated by extreme value theory and are therefore primarily justified in the tail of the distribution. As a result, their adequacy may be less accurate in the earlier stages of the procedure, where the observations do not yet correspond to the extreme regime. This mismatch may induce estimation errors in the first iterations, which can propagate through the sequential updates and affect the final estimate. Although the paper outlines possible extensions toward more flexible modeling strategies, a deeper investigation of model misspecification—especially outside the extreme region—and its impact on the procedure would be a valuable direction for future work.

Beyond the specific results obtained in this work, the proposed methodology also highlights an interesting statistical perspective. The sequential nature of the procedure, together with the use of a primary estimation criterion, naturally suggests the introduction of a second statistical criterion aimed at validating the estimates at each stage.

In particular, incorporating a cross-validation-type phase within the sequential design provides an additional layer of assessment for the stability and reliability of the estimated parameters. This idea, which emerges from the structure of the method itself, opens a promising direction for future research and would require further theoretical investigation.

## Figures and Tables

**Table 1:** Results obtained using the *Staircase* method through simulations under the exponential model.

Relative Error			
On the Parameter		On $s_{\alpha }$	
Mean	Std	Mean	Std
−0.252	0.178	0.4064874	0.304

**Table 2:** Results obtained using the *Staircase* method through simulations under the Gaussian model.

Relative Error					
On μ		On σ		On $s_{\alpha }$	
Mean	Std	Mean	Std	Mean	Std
−0.059	0.034	1.544	0.903	−1.753	0.983

**Table 3:** Results obtained through *CRM* on simulations for the exponential model.

Relative Error			
On the 0.1-Quantile		On the $10^{-3}$-Quantile	
Mean	Std	Mean	Std
0.129	0.48	−0.799	0.606

**Table 4:** Estimation of the (1−α)-quantile, ${\tilde{s}}_{\alpha }=469.103$, through procedure Section 4.5 with K=50.

Minimum	Q25	Q50	Mean	Q75	Maximum
67.07	226.50	327.40	441.60	498.90	10,320.00

**Table 5:** Estimation of the (1−α)-quantile, ${\tilde{s}}_{\alpha }=469.103$, through procedure Section 4.5 for different values of *K*.

	${\tilde{s}}_{m}$ for K=30		${\tilde{s}}_{m}$ for K=50	
${\tilde{s}}_{\alpha }$	Mean	Std	Mean	Std
469.103	1276.00	12,576.98	441.643	562.757

**Table 6:** Mean and std of relative errors on the (1−α)-quantile of GPD calculated through 400 replicas of Procedure 2.

Parameters	Relative Error on ${\tilde{s}}_{\alpha }$	
	Mean	Std
c=0.8, $a_{0}=1.5$ and ${\tilde{s}}_{\alpha }=469.103$	−0.222	0.554
c=1.5, $a_{0}=1.5$ and ${\tilde{s}}_{\alpha }=31,621.777$	−0.504	0.720
c=1.5, $a_{0}=3$ and ${\tilde{s}}_{\alpha }=63,243.550$	0.310	0.590

**Table 7:** Mean and std of the relative errors on the 1−α-quantile of GPD on complete and binary data for samples of size n=250, computed through 400 replicas of both estimation procedures. Estimates on complete data are obtained with de Valk’s method; estimates on binary data are provided by the sequential design.

	Relative Error on the (1−α)-Quantile			
Parameters	on Complete Data		on Binary Data	
	Mean	Std	Mean	Std
c=0.8, $a_{0}=1.5$ and $s_{\alpha }=469.103$	0.052	0.257	−0.222	0.554
c=1.5, $a_{0}=1.5$ and $s_{\alpha }=31,621.777$	0.086	0.530	−0.504	0.720
c=1.5, $a_{0}=3$ and $s_{\alpha }=63,243.550$	0.116	0.625	0.310	0.590

**Table 8:** Mean and std of relative errors on the (1−α)-quantile of Weibull distributions on complete and binary data for samples of size n=250 computed through 400 replicas. Estimates on complete data are obtained with de Valk’s method; estimates on binary data are provided by the sequential design.

	Relative Error on the (1−α)-Quantile			
Parameters	on Binary Data		on Complete Data	
	Mean	Std	Mean	Std
$a_{0}=3$, $b_{0}=0.9$ et $s_{\alpha }=25.69$	0.282	0.520	0.127	0.197
$a_{0}=3$, $b_{0}=1.5$ et $s_{\alpha }=10.88$	−0.260	0.490	0.084	0.122
$a_{0}=2$, $b_{0}=1.5$ et $s_{\alpha }=7.25$	−0.241	0.450	0.088	0.140

## Data Availability

The original contributions presented in this study are included in the article. Further inquiries can be directed to the corresponding author.
